# Successful lung-preserving tractotomy to treat intrathoracic wood impalement after high-fall trauma: a case report

**DOI:** 10.1186/s12245-026-01310-1

**Published:** 2026-07-08

**Authors:** Yugo Wakayama, Tetsuya Fukuta, Haruna Makigano, Yoshinori Kakino, Hirotaka Asano, Soichiro Kano, Erika Takada, Takahito Miyake, Shozo Yoshida, Hideshi Okada

**Affiliations:** Advanced Critical Care Center, Gifu University Hospital, 1-1 Yanagido, Gifu- city, Gifu-Ken, 501-1194 Japan

**Keywords:** Thoracic impalement, Wooden foreign body, Penetrating trauma, Tractotomy, Lung-sparing surgery, Fall injury, Case report

## Abstract

**Background:**

Thoracic impalement injuries caused by wooden objects are extremely rare and present unique diagnostic and therapeutic challenges owing to the radiolucent nature of wood and the risk of organic contamination. Lung-preserving tractotomy is a valuable, but infrequently reported technique for managing penetrating lung trauma caused by wooden foreign bodies.

**Case presentation:**

A 39-year-old healthy arborist fell from a height of approximately 20 m during tree felling. On presentation, the patient was hemodynamically stable, with a small anterior chest wound and subcutaneous emphysema. Chest computed tomography (CT) revealed a 24 × 3 cm wooden foreign body completely embedded within the left lung, along with rib fractures and a moderate hemothorax. No vascular extravasation was observed. Emergency anterolateral thoracotomy was performed to avoid the risk of vascular injury and organic contamination. However, the wood fragments were firmly wedged between the ribs, rendering axial extraction impossible. Therefore, the object was exposed and safely removed using stapler-assisted tractotomy. Broad-spectrum antibiotics were administered, and the patient recovered uneventfully. He was discharged on postoperative day 18 and returned to full activity without complications. Follow-up CT at 8 months revealed complete resolution without evidence of empyema or atelectasis.

**Conclusions:**

This case underscores the importance of comprehensive CT evaluation, avoidance of field extraction, and aggressive infection control in thoracic impalement injuries involving wooden objects. Tractotomy is an effective lung-preserving strategy that facilitates safe removal of deeply embedded wooden foreign bodies while avoiding the need for major pulmonary resection.

## Background

Extremely rare impalement injuries represent approximately 0.14% of all trauma cases and typically occur in high-energy mechanisms such as falls from high places and industrial accidents.

Thoracic impalement by wooden objects is particularly uncommon and presents unique diagnostic and therapeutic challenges. Because wood has low radiodensity and variable attenuation depending on its moisture content, it may be overlooked on imaging [1–3]. Organic contamination carries a high risk of infection and requires appropriate antimicrobial therapy and meticulous debridement [1–3, 5, 6, 13].

Removal of penetrating foreign bodies in the field is contraindicated as it may precipitate fatal hemorrhage or hemodynamic collapse; therefore, current guidelines recommend controlled extraction after arrival at a trauma facility. Exclusion of cardiac and great vessel injuries and evaluating the routes of penetration are critical when evaluating penetrating thoracic trauma. Contrast-enhanced CT with three-dimensional reconstruction is valuable for trajectory assessment and surgical planning.

Preservation of lung function, hemorrhage control, and infection prevention are key principles in the management of penetrating lung injuries. Wedge resection and lobectomy have historically been the main surgical approaches; however, tractotomy allows direct visualization of injured bronchovascular structures by opening the wound tract and facilitates lung preservation [7–10]. Although tractotomy has been reported in metallic chest impalement injuries, reports involving completely embedded wooden foreign bodies remain scarce [2, 6, 11].

We describe a case of intrathoracic wooden impingement resulting from a 20-meter fall that was successfully managed with emergency tractotomy. The foreign body was safely removed with preservation of lung parenchyma, and a favorable outcome was achieved.

## Case presentation

A healthy 39-year-old man working as an arborist fell from a height of approximately 20 m when part of his dual-harness support system remained attached to a tree section to be felled. This section likely struck the surrounding branches during descent, partially dissipating kinetic energy.

**Fig. 1:**
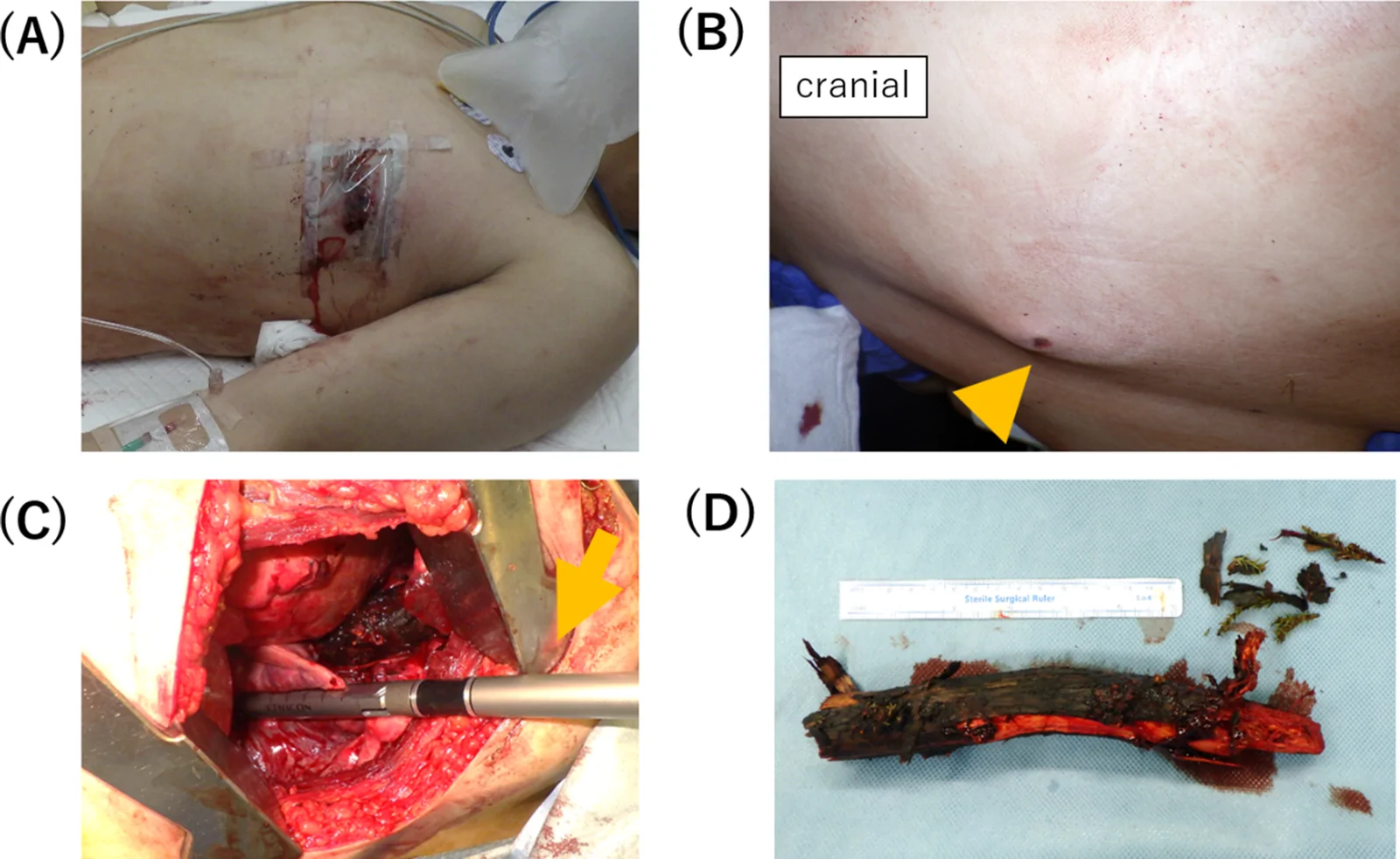
Status of patient upon arrival. (**A**) Small puncture wound on the left anterior chest with mild subcutaneous emphysema. (**B**) Localized swelling on the left posterior chest without visible external foreign bodies. (**C**) Non-contrast CT image of a wooden foreign body lodged in the left lung and a moderate hemothorax. (**D**) 3D reconstruction reveals complete intrathoracic embedding of the wooden fragment

At the scene, a small open wound with air leakage was noted on the left anterior chest, and a three-sided occlusive dressing was applied (Fig. 1A and B). The patient remained hemodynamically stable and was transported to our facility by an emergency helicopter.

On arrival, he was alert (Glasgow Coma Scale score, 15) with stable respiratory and circulatory status. An initial survey revealed a small anterior chest wound with mild subcutaneous emphysema, without paradoxical chest movement, jugular venous distention, or breath-sound asymmetry, and no visible foreign object.

The second survey revealed localized swelling and tenderness over the left posterior chest without any externally visible foreign body. Arterial blood gas and laboratory test results revealed leukocytosis and elevated creatine kinase, fibrin degradation products, and D-dimer levels, without coagulopathy or metabolic abnormalities. (Table 1)

**Table 1:** Laboratory test results

< CBC>			< Biochemistry>		
White Blood Cells	28,960	/uL	Total Protein	5.9	g/dL
Neutrophil	82.6	%	Albumin	3.9	g/dL
Monocyte	4.6	%	Creatinine Kinase	300	g/dL
Lymphocyte	12.0	%	Aspartate Transaminase	28	IU/L
Eosinophil	0.6	%	Alanine Transaminase	24	IU/L
Basophil	0.2	%	Alkaline Phosphatase	61	IU/L
Atypical Lymphocyte	0	%	γ-Glutamyl Transpeptidase	15	IU/L
			Creatinine	0.78	mg/dL
Red Blood Cells	4.45 × 10^6^	/uL	Blood Urea Nitrogen	12.8	mg/dL
Hemoglobin	12.9	dL	Total Bilirubin	0.5	mg/dL
Hematocrit	37.6	%	Sodium	139	mEq/L
Platelet	26.2 × 10^4^	uL	Potassium	3.6	mEq/L
**< Coagulation Status>**			Chlorine	106	mEq/L
APTT	22.1	sec	Calcium	8.0	mg/dL
PT-INR	10.96		Phosphorus	4.1	mg/dL
Fibrinogen	219	mg/dL	Magnesium	1.8	mg/dL
D-dimer	4.4	ug/mL	C-reactive protein	0.04	mg/dL
FDP	10.8	ug/mL	Blood Glucose	163	mg/dL
Antithrombin III	97	%			
**< Arterial Blood Gas>**					
F_I_O_2_	1.0				
pH	7.350				
PaCO_2_	45.3	mmHg			
PaO_2_	144.0	mmHg			
HCO_3_^−^	27.2	mmol/L			
Base Excess	-0.9				
Lactate	10	mg/dL			

**Fig. 2:**
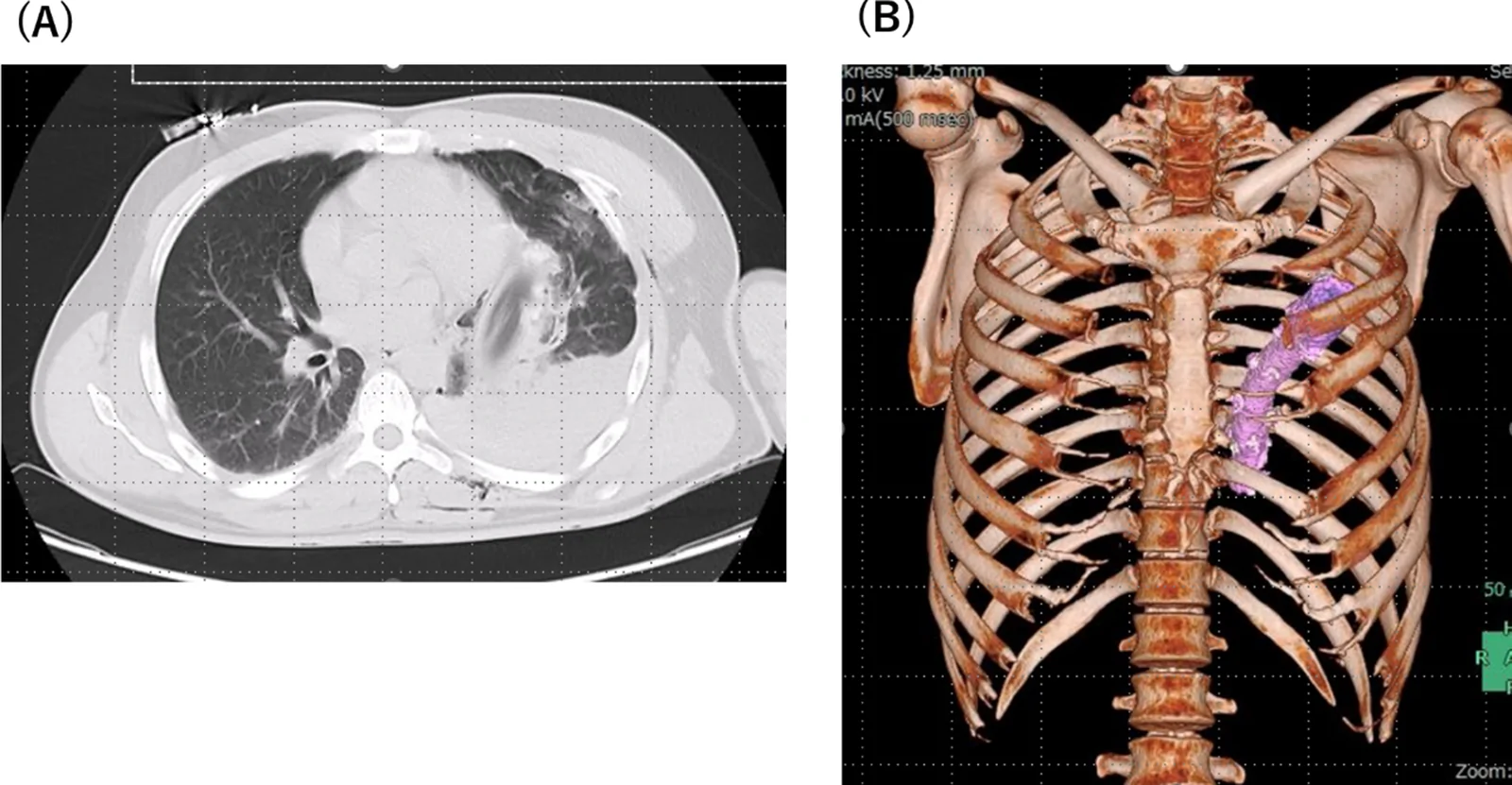
Surgical findings. (**A**) An anterolateral thoracotomy reveals a wooden fragment firmly lodged within the thoracic cavity, traversing the intercostal space. Penetration tract opened using stapler-assisted tractotomy. (**B**) Extracted 24 × 3 cm wooden fragment with attached plant and soil debris, indicating organic contamination

Chest computed tomography (CT) revealed a 24 × 3 cm wooden foreign body completely embedded in the left lung, accompanied by fractures of the left anterior fourth and fifth ribs, a fracture of the left posterior ninth rib, pulmonary contusion of the left upper lobe, and a moderate hemothorax (Fig. 2A and B). Contrast-enhanced CT revealed no vascular extravasation or injury involving the heart or great vessels. Three-dimensional reconstruction clarified the discrepancy between the skin puncture site and the internal trajectory, explaining the absence of externally visible wooden fragments. No other major traumatic injuries were identified on the initial trauma evaluation.

We performed an anterolateral thoracotomy considering the risk of vascular injury and organic contamination (Fig. 1C). The patient was positioned supine with a slight anterior tilt of the left hemithorax to prepare for a possible massive intraoperative hemorrhage, and the left hemithorax was selected. Upon entering the chest through the fifth intercostal space, the wooden fragment penetrates from the inferior margin of the upper lobe to the central portion of the lower lobe. It was firmly wedged between the ribs to prevent extraction along the penetration axis. Tractotomy was then performed because deep bronchovascular structures were difficult to evaluate. A linear surgical stapler (ECHELON FLEX^®^ Powered Plus Stapler and ECHELON^®^ ENDOPATH™ 60 mm Reloads with Gripping Surface Technology (Black cartridge) (Ethicon Endo-Surgery, LLC., Cincinnati, OH, USA) was applied along the trajectory of the wooden object to divide the lung parenchyma. The foreign body was safely removed (Fig. 1D), and bronchovascular injury or persistent air leakage was identified in the open tract. The thoracic cavity was extensively irrigated with large volumes of warm normal saline until no visible contamination remained, and all residual wood fragments and plant debris were meticulously removed to minimize the risk of infectious complications.

**Fig. 3:**
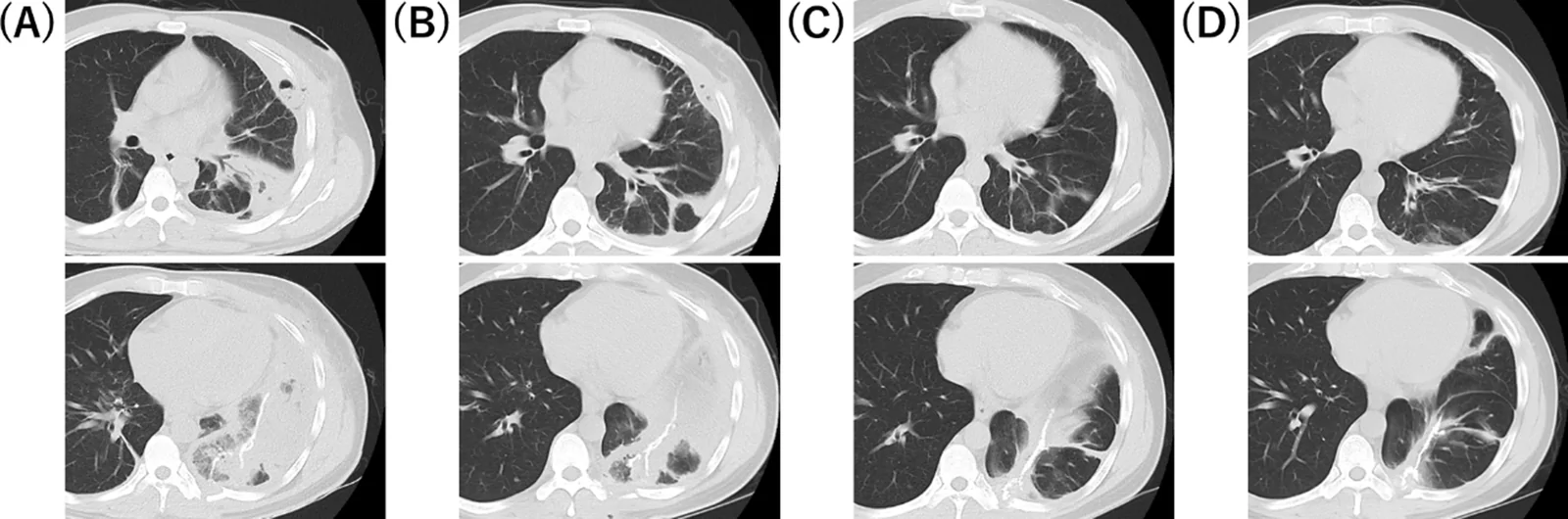
Postoperative computed tomography findings. (**A**) 1 week, (**B**) 4 weeks, (**C**) 16 weeks, and (**D**) 32 weeks after surgery, demonstrating gradual resolution of ground-glass opacities without empyema, atelectasis, or pneumothorax

Postoperative prophylaxis involved piperacillin–tazobactam and gentamicin for 14 days, followed by oral amoxicillin-clavulanate for 14 days. The patient was extubated on postoperative day (POD) 2, underwent chest tube removal on POD 3, and was discharged without complications on POD 18. Follow-up CT at 1, 4, 16, and 32 weeks confirmed total resolution of ground-glass opacities without atelectasis or empyema. (Fig. 3). At one year after the injury, CT confirmed rib nonunion without any evidence of recurrent infection. The patient had no functional limitations in daily life and returned to full-time work.

## Discussion

Penetrating thoracic injuries caused by wooden foreign bodies present unique diagnostic and therapeutic challenges. Wood is radiolucent, and its CT attenuation varies with moisture content, which may lead to underestimation of the depth and trajectory during initial evaluation [1–3]. Despite the small external wound in our patient, the wooden fragment was completely embedded within the thoracic cavity and could not be reliably identified on plain radiography. Contrast-enhanced CT with 3D reconstruction is indispensable for determining penetration routes and planning surgical access [2, 8]. Historically, tractotomy was introduced as a lung-sparing alternative to lobectomy for penetrating pulmonary trauma [7]. Although it was initially applied mainly to gunshot and stab injuries [8, 10], recent reports have demonstrated its usefulness in selected impalement injuries, particularly when preservation of pulmonary parenchyma and direct visualization of injured bronchovascular structures are desirable [3, 8, 11].

Foreign body extraction at the scene of injury is contraindicated because the object may serve as a tamponade; uncontrolled removal can precipitate catastrophic hemorrhage or circulatory collapse. Adherence to the principle of “do not remove in the field” and prompt transport to a trauma-capable center are essential for survival [5, 6, 12, 13].

Wooden foreign bodies carry a high risk of bacterial and fungal contamination, and retained plant material may result in both early postoperative infection and delayed complications such as empyema or lung abscess. Therefore, early administration of broad-spectrum antimicrobial therapy combined with extensive irrigation and meticulous debridement is central to preventing infectious complications [2, 6, 13].

Preservation of lung parenchyma after penetrating lung trauma is critical for postoperative respiratory function. Previous studies have demonstrated that lung-sparing techniques, including tractotomy, are associated with better postoperative outcomes than anatomical lung resection in appropriately selected patients [14]. When extraction along the penetration axis is difficult or when deep hilar structures require visualization, tractotomy is advantageous because it opens the wound tract, provides direct visualization of bronchovascular structures, and allows targeted hemostasis and repair [7–10]. Compared with anatomical lung resection such as lobectomy, tractotomy minimizes unnecessary parenchymal loss, which is particularly beneficial in otherwise healthy lungs [7, 8, 14]. In the present case, the wooden fragment was firmly impacted between the ribs and could not be removed safely along the original trajectory. **Forceful extraction along the original trajectory could have resulted in additional pulmonary laceration**,** uncontrolled hemorrhage**,** disruption of bronchovascular structures**,** and retention of contaminated wooden fragments.** Stapler-assisted tractotomy enabled controlled exposure of the wound tract, safe removal of the foreign body, direct assessment of bronchovascular injuries, and preservation of both lung lobes. Combined with extensive lavage and prolonged antimicrobial therapy, this approach resulted in an uneventful recovery and excellent long-term pulmonary function [2, 7, 8, 10].

Favorable outcomes of wooden thoracic impalement depend on avoiding field extraction, precise preoperative CT-based trajectory assessment, aggressive infection control, and lung-preserving surgery, such as tractotomy.

To summarize, wooden thoracic impalement injuries require avoidance of field extraction, precise CT evaluation, and aggressive infection control. Tractotomy enables safe removal and lung preservation and is thus a valuable strategy for achieving favorable outcomes in rare but high-risk injuries involving wood.

## Data Availability

The datasets used and analyzed during the current study are available from the corresponding author upon reasonable request.
